# Annotations

**Published:** 1901-06-22

**Authors:** 


### Annotations.

### Consulting-Room Ethics.

What is called medical etiquette is mostly founded upon the good old maxim that one should do as one would be done by, and there can be no doubt that by obedience to its rules harmony within the profession is much promoted. Those outside, however, are apt to look at the matter in a somewhat different light, and many good people are to be found who object most strongly to any limitation being placed upon their right to consult just whomsoever they like without explanation or apology. One of the rules, however, on which medical men in all countries lay the greatest stress is that, except in cases of emergency, no man shall take or prescribe for another man's patient. In other words, he must satisfy himself that the other man is "off" before he himself goes "on." So far as visiting a patient this rule still holds good, and is generally obeyed, much to the advantage of all parties. But of late years considerable laxity has grown up in regard to consulting-room practice; many men, and more particularly the specialists, holding that they can properly be consulted by anyone who offers them a fee. All this is very contrary to the strict etiquette of old days, but in excuse of it there is a good deal to be said. The growth of specialism is really at the root of the change. In former times the patient who went to a consultant went for advice which had to be carried into effect by the general practitioner. Nowadays the consultant or more properly the specialist, must himself carry out what he recommends. His advice that a certain thing should be done hinges upon his special knowledge how to do it, and he feels that he would be dealing absolutely dishonestly with his patient were he to send him back to a man of whose skill he knows nothing for the carrying out of a treatment which would be useless unless skilfully performed. This feeling is, we have no doubt, at the bottom of many of the actions for which specialists are continually blamed. Then one must remember that the general practitioner does not always show the sweetest side of his nature when he finds that his pet patient has gone off to consult someone else; so that, for many reasons, the "consultant," if a man of peace, is tempted to take the patients Providence provides, and ask no questions as to their previous medical attendance. At any rate, this is what is done every day. The Conseil Général des Sociétés d'Arrondissement of Paris has recently been laying down laws for the guidance of its members, and has among other things adopted the principle that "a consulting-room is neutral ground where a medical man may give advice without any regard to the medical man who usually attends the patient." This is putting the matter rather strongly, and such a rule will certainly raise protests from many. But is it not in accordance with the facts of life?

### The Royal College of Surgeons.

The Fellows of the Royal College of Surgeons of England will, on July 4th, proceed as usual to the election of three of their number to fill vacancies on the Council. These are caused by the retirement in rotation of Mr. Macnamara, Mr. Mayo Robson, and Mr. Watson Cheyne. Both Mr. Mayo Robson and Mr. Watson Cheyne offer themselves for re-election and we can hardly doubt that they will be appointed, and, as there are plenty of good candidates for the odd post, the Council of the Royal College will remain, as always, thoroughly representative of good surgery. Some may ask, "What more can we want?" But there are others who feel that there is something beyond surgery, and that the governing body of a College which has on its roll some 20,000 members, ought to take a much stronger position in regard to the maintenance of professional discipline than the Council of the College of Surgeons has so far shown itself ready to do. To people who are impressed with this view of the situation all the discussion which is current at the present time as to the proper distribution of representation among the various medical schools seems sadly beside the mark. They would have opinions on medical politics made the touchstone rather than position in medical schools. At present the tendency is to push on to the General Medical Council—a body which is most ill supplied with weapons for the purpose—the whole duty of maintaining discipline in the profession; except in extreme cases, the licensing bodies stand aside. Many of us, however, think that those bodies which have the power have also the duty laid upon them of exercising a far greater restraining influence upon their members than they have hitherto put in operation. The election of men merely eminent in surgery, while adding brilliance to the coterie, leaves untouched the problems which trouble the profession.

### A Great Experiment in Hygiene.

Last Saturday saw the inauguration of a great practical experiment in scientific hygiene. As the result of a long series of investigations the source and origin of malarial infection has been traced to the malaria parasite, and the whole history of this parasite has been laid bare. This was a great achievement. The pæans of science have been sung from many platforms, and great has been the acclaim at its unearthing this mysterious disease, a disease by which man is barred from the full utilisation of perhaps half the otherwise habitable globe. But with knowledge comes responsibility. The wondrous tale of the malaria parasite would be of only indirect importance to mankind were it not that there is hope that when its lessons are applied they may result in saving life and adding to the wealth and happiness of nations. And this is the experiment which is going to be tried; the practical application of the knowledge we possess. We know as the result of the experiments conducted last year in the Campagna by the delegates of the London School of Tropical Medicine, that it is possible by protecting oneself from mosquito bites to keep free from malaria. What Dr. Ross and Dr. Logan Taylor, the delegates of the Liverpool school, who last Saturday sailed out from that city, wish to show is that by ridding a town of mosquitos the same thing can be done for all the inhabitants. They go out with funds sufficient to employ 30 to 40 natives for a year, and their aim is to clear Freetown of mosquitos by filling up the puddles and emptying the tanks of water in which they breed, and by fumigating the houses in which they lodge they hope to put a check to the multiplication of the mosquito and thus to break up the cycle of the malaria parasite's life by eliminating the creature in whose body one phase of that cycle must be passed. This once done the municipal authorities must continue the work. The experiment will go on for 10 years, but long before then we may hope to see some results in the diminished prevalence of this fell disease.

